# Introduction to Trial‐Level Subgroup Analysis: A Tutorial

**DOI:** 10.1002/cesm.70100

**Published:** 2026-08-03

**Authors:** Peter J. Godolphin, Conor D. Tweed, David J. Fisher

**Affiliations:** ^1^ UCL Innovative Clinical Trials Unit, Institute of Clinical Trials and Methodology University College London London UK

## Abstract

This tutorial introduces the concept of trial‐level subgroup analysis in meta‐analysis. We describe the rationale for conducting subgroup analyses, distinguish trial‐level from participant‐level characteristics, outline how to properly interpret effects within subgroups, and illustrate how to perform a trial‐level subgroup analysis, including formal statistical testing for differences between subgroups. A complementary micro‐learning module is provided to support additional learning of these concepts.

## 1 Introduction

A clinical trial is designed to assess the effect of a treatment on average across all included participants and is powered accordingly [1]. However, trial investigators are often interested in whether the treatment effect varies according to specific patient characteristics, thereby assessing the presence of effect modification [2]. Identifying potential effect modifiers is usually guided by prior clinical understanding, biological plausibility, or evidence from previous studies.

For example, if in a hypertension trial there is rationale for a blood pressure‐lowering drug to be more effective in younger patients, investigators may wish to investigate whether age modifies the treatment effect. In this hypothetical example, trial participants could be split into two groups: those less than 50 years of age at the time of randomization, and those greater than or equal to 50. The treatment effect can be estimated separately within each subgroup, accompanied with a formal statistical test to determine whether these subgroup effects differ. This provides statistical evidence about whether there is *heterogeneity* in the effect of the blood pressure‐lowering drug based on this dichotomy of patients age.

## 2 Interpreting Subgroup Effects in a Single Trial

We begin with a single‐trial example, which will form the foundation for trial‐level subgroup analyses within a meta‐analysis. We continue the hypothetical example from the Introduction, but with additional information, summarized in Table 1.

**Table 1:** Results from a hypothetical hypertension trial where the outcome of interest is mortality.

Hypothetical trial	Control (*n*/*N*)	Treatment (*n*/*N*)	Risk ratio (95% CI)
All participants	128/380	93/380	0.73 (0.58, 0.91)

*Note: N* refers to the number of participants randomized; *n* refers to number of events (deaths).

**Figure 1:**
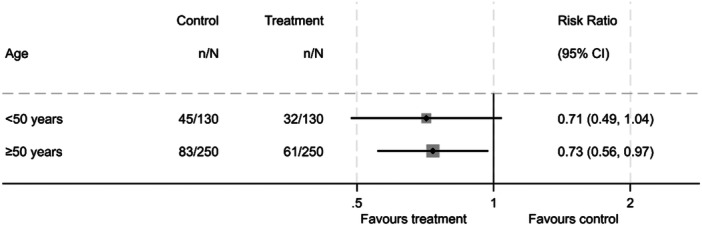
Subgroup analysis by age in the hypothetical hypertension trial.

Consider a subgroup analysis based on age. In this hypothetical trial, assume that 260 participants (across both randomized arms) were younger than 50, and 500 were 50 or older. We can calculate the treatment effect (here using a risk ratio) in the < 50 years subgroup, and the treatment effect in the ≥ 50 years subgroup, with their 95% confidence intervals (Figure 1).

In the ≥ 50 years subgroup, the risk ratio (RR = 0.73) is very similar to the overall risk ratio (Table 1), showing evidence of a benefit of the blood pressure‐lowering drug. In the < 50 years subgroup the risk ratio is also similar in size (RR = 0.71), again indicating benefit. However, the 95% confidence interval crosses the line of no effect, and so the *p*‐value associated with this subgroup will be greater than conventional levels for statistical significance (*p* > 0.05). Importantly, this does not mean that we have evidence that the intervention is not beneficial in this subgroup. Rather, this subgroup is underpowered to detect an effect of this magnitude. Absence of statistically significance should not be interpreted as evidence of no effect.

Consider the implications of this subgrouping. The trial has been split into two, one larger group with 500 participants and one much smaller group with only 260 participants. The smaller group has considerably less information, and so the confidence interval around this subgroup effect should be expected to be wider. In this example, it happens to be wide enough that it crosses the line of no effect.

It is important to understand that if a trial was designed to test an overall treatment effect, then any subgrouping must lead to a reduction in sample size in any given subgroup, and hence the width of the confidence interval will increase. Even in the larger subgroup (≥ 50 years) the confidence interval is wider than for the overall effect. This point is illustrated vividly in an editorial from Spears et al. [3].

## 3 Test of Subgroup Differences Within a Single Trial

As the previous example illustrates, interpreting statistical significance *within* individual subgroups is insufficient for determining whether the treatment effects differ meaningfully *between* subgroups. Instead, we need a formal statistical test against the null hypothesis that the effect of the blood‐pressure lowering drug in the < 50 years subgroup is *equal* to that in the ≥ 50 years subgroup. Such a test (often referred to as a “test for interaction,” and implemented as a Wald test or likelihood ratio test) evaluates whether the treatment effects differ between subgroups. In this hypothetical trial, the test compares the difference in log risk ratios to its standard error. A small *p*‐value provides evidence against the null hypothesis. In this example, the *p*‐value from the Wald test is 0.89, providing no evidence that the effect of the blood‐pressure lowering drug differs between age subgroups. Because of the low power typically available for interaction tests, alpha levels are sometimes raised from 5% to 10% (so that *p* < 0.10 is considered statistically significant), although this should be agreed in advance. This testing procedure and its interpretation is fundamental to correctly carrying out subgroup analyses.

## 4 Participant‐Level and Trial‐Level Characteristics

When considering systematic reviews and meta‐analysis we have information from multiple trials. This means that we can explore heterogeneity in *two* ways rather than just one:

1. Whether a *trial*‐level characteristic is causing heterogeneity (trial‐level subgroup analysis).
2. Whether a *participant*‐level characteristic is causing heterogeneity (participant‐level subgroup analysis).

First, it is useful to clarify the distinction between “trial‐level” and “participant‐level” characteristics. A “trial‐level” characteristic is one that doesn't vary within any single trial (i.e., each trial is described by just a single value of the characteristic) but may vary *between* the trials in the meta‐analysis. Typical examples include trial design features such as whether the trial is individually randomized or cluster randomized [4] and properties of the intervention, such as dose level. By contrast, participant‐level characteristics vary *within* a trial: each *participant* is described by a value of the characteristic (e.g., their age). Note that in some circumstances, a characteristic may have both trial‐level and participant‐level features or may be considered “trial‐level” in one context but “participant‐level” in another. For example, characteristics such as disease severity may be constant in one trial but vary within another trial, depending on trial eligibility criteria. In the remainder of this article, we will assume that such circumstances do not apply, and instead that all trials may be classified straightforwardly into distinct values of the trial‐level characteristic of interest.

Participant‐level subgroup analyses are less common in meta‐analysis due to the required data often being less available and analysis being more complex due to the potential for aggregation bias (for more details see, e.g., Refs. [5, 6, 7]). This tutorial therefore focuses exclusively on trial‐level subgroup analysis.

## 5 Trial‐Level Subgroup Analysis

**Figure 2:**
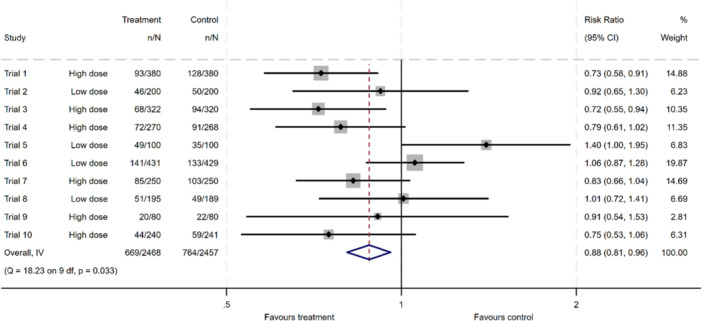
Hypothetical meta‐analysis for an outcome of mortality.

Now consider that this hypothetical trial is 1 of 10 evaluating the same blood‐pressure lowering drug, with all of these trials identified in a systematic review. Figure 2 shows the forest plot from an inverse‐variance common‐effect meta‐analysis for the same outcome of mortality. In this example, there is evidence of heterogeneity in the overall treatment effects between trials (heterogeneity *p*‐value = 0.033).

Note that this tutorial uses common‐effect meta‐analysis for illustration. In practice, the choice between common‐effect, fixed‐effects and random‐effects models should be based on the research question and the plausibility that all trials share a common true effect. Random‐effects models may be more appropriate when heterogeneity is expected, though this complicates the interpretation of subgroup analyses.

Analogous to the single‐trial setting, the evidence can be split into groups. Here, instead of grouping the participants, the trials are grouped; four Low‐dose trials and six High‐dose trials.

**Figure 3:**
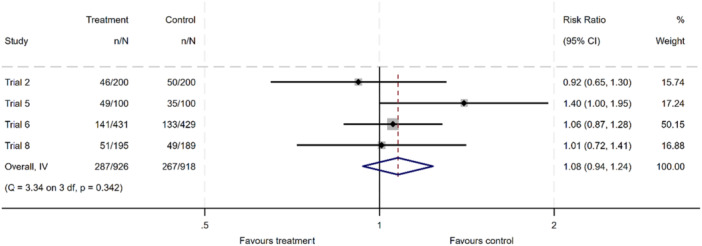
Meta‐analysis of the Low‐dose trials.

A meta‐analysis of the four Low‐dose trials gives a risk ratio of 1.08 (95% CI 0.94, 1.24; Figure 3 The heterogeneity (Cochran's *Q*) statistic is 3.34, which when compared to a chi‐square distribution with 3 degrees of freedom (as there are four trials), gives a heterogeneity *p*‐value of 0.342. So, within the Low‐dose trials, statistical evidence of heterogeneity has not been clearly identified.

**Figure 4:**
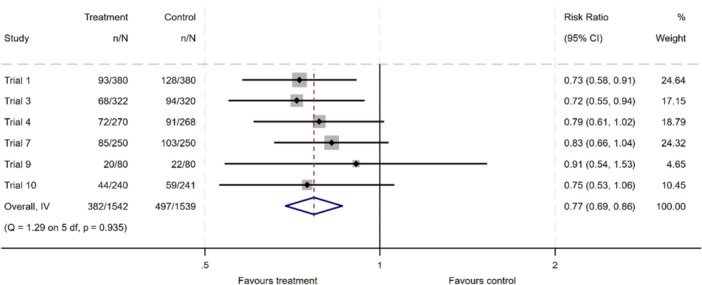
Meta‐analysis of the high‐dose trials.

Following the same approach for the High‐dose trials gives a risk ratio of 0.77 (95% CI 0.69, 0.86; Figure 4). The heterogeneity statistic for this subgroup is 1.29, which when compared to a chi‐square distribution with 5 degrees of freedom (as there are six trials), gives a heterogeneity *p*‐value of 0.935. So, again, statistical evidence of heterogeneity has not been identified.

Splitting the trials into these two subgroups may have explained the heterogeneity that was identified when they were all considered together. However, it is important not to interpret these subgroup effects without first considering a test of subgroup differences.

## 6 Test of Subgroup Differences in a Meta‐Analysis

There are various ways to formally test for subgroup differences in meta‐analysis, but here we provide a simple approach that uses Cochran's *Q* statistics for heterogeneity, but applied across subgroups rather than between individual studies. This approach is used by default in RevMan, and in other common statistical software packages, such as metan in Stata [8]. Other approaches are discussed elsewhere [9, 10].

This approach splits the total amount of heterogeneity ($Q_{T}$) identified across all trials into the amount of heterogeneity identified *within* the subgroups ($Q_{W}$) and the amount that is explained by differences *between* the subgroups ($Q_{B}$). For a trial‐level subgroup analysis with S subgroups, we have:

$$Q_{T}=Q_{B}+Q_{W},\text{where}\;Q_{W}=\sum_{i=1}^{S}Q_{i}$$

To work out the test for subgroup differences, first $Q_{B}$ is calculated, which is compared to a chi‐square distribution with S−1 degrees of freedom. As before, the null hypothesis is that there is no heterogeneity between the subgroups, and so small *p*‐values (typically < 0.1) provide evidence against this.

In the example, $Q_{T}$ is the *Q* statistic from the overall meta‐analysis (Figure 2). With two subgroups (Low‐dose and High‐dose), S=2, and $Q_{W}=Q_{1}+Q_{2}$, where $Q_{1}$ is the *Q* statistic from the Low‐dose subgroup (Figure 3) and $Q_{2}$ is the *Q* statistic from the High‐dose subgroup (Figure 4).

$Q_{W}=3.34+1.29=4.63$, and, from Figure 2, $Q_{T}=18.23$. Therefore, $Q_{B}=18.23-4.63=13.60$, and upon comparing this value to a chi‐square distribution with 1 degree of freedom (as S−1=2−1=1) the *p*‐value for the test of subgroup differences is 0.00023. This provides strong evidence to reject the null hypothesis, suggesting that there is heterogeneity between the two subgroup effects, i.e., that in this meta‐analysis the identified heterogeneity can be explained by dose, suggesting that the effect of the blood‐pressure lowering drug works differently at a Low and High dose.

**Figure 5:**
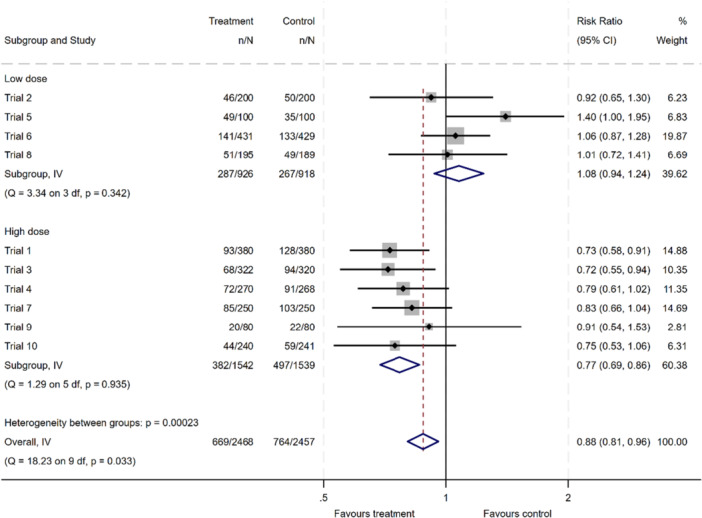
Trial‐level subgroup analysis by dose.

The two subgroup effects, combined with the significant test for subgroup differences, suggests that the treatment benefit may be greater at High doses compared to Low doses. This interpretation is only valid because there was statistical evidence of subgroup differences. When reporting trial‑level subgroup analyses, authors should describe both the subgroup effect estimates and the test for subgroup differences. This information is summarized in Figure 5.

However, caution is warranted. First, this trial‐level subgroup analysis may be confounded by other trial (or patient) level characteristics that may differ between the Low‐dose and High‐dose trials. The observed difference might be due to dose itself, but could instead be due to other systematic differences between these trial groups. Second, the categorization of trials into Low‐ and High‐dose groups is dependent on the chosen dose thresholds. Alternative thresholds may yield different subgroup allocations and estimates, highlighting the risks of dichotomizing continuous variables. Note that if no evidence of subgroup differences is found, the individual subgroup effects should not be interpreted in isolation, and instead interpretation should remain with the overall meta‐analysis pooled estimate.

A follow‐on tutorial [11] provides key tips to avoid making common errors when conducting trial‐level subgroup analysis.

## 7 Further Reading and Online Content

More information on trial‐level subgroup analysis can be found in Chapter 10.11 of the Cochrane Handbook for Systematic Reviews of Interventions [12] and a tutorial by Kanellopoulou et al. [13]. A follow‐on tutorial explores trial‐level subgroup analysis for more than two categories, how to handle continuous trial‐level characteristics and when and how to use meta‐regression [11]. Cochrane Training has produced a micro‐learning module covering trial‐level subgroup analysis to accompany both tutorials [10].

## Author Contributions

**Peter J. Godolphin:** conceptualization, formal analysis, writing – original draft, writing – review and editing. **Conor D. Tweed:** conceptualization, writing – review and editing. **David J. Fisher:** conceptualization, writing – review and editing.

## Conflicts of Interest

The authors declare no conflicts of interest.

## Peer Review

The peer review history for this article is available at https://www.webofscience.com/api/gateway/wos/peer-review/10.1002/cesm.70100.

## Supporting information


Supporting File

## Data Availability

All data used in this article is aggregate data and is “hypothetical.” This can be extracted from Table 1, Figures 1 and 2.
