# Supplementary material for: Introduction to Trial‐Level Subgroup Analysis: A Tutorial
Source: Cochrane Evid Synth Methods. 2026 Aug 3;4(5):e70100. doi: 10.1002/cesm.70100 (PMC13431748; doi:10.1002/cesm.70100)
Supplement: Supplementary file 1 — Supporting File [file CESM-4-e70100-s001.docx]

**Sketch for micro-learning module for CESM tutorials on subgroup analysis:**

A single micro learning module to cover the “Introduction to subgroup analysis” and “Exploring heterogeneity in meta-analysis using trial-level characteristics” tutorials, with the following parts:

1. **Are factors patient-level, trial-level, or could be both?**
   1. A short-length video (approx. 3-5 mins)
      - Explaining the difference between trial-level and patient-level factors, based on whether a factor would typically be associated with an individual trial or with an individual participant. Brief mention of issues arising if factor might be both (e.g. mean age)
      - Explaining why this is important – splitting trials into subgroups by a trial-level factors means we still have sets of trials, splitting trials into subsets (via a patient-level factor) means that we have parts of trials/non-randomised comparisons etc.
      - Describing analyses that you could use to explore heterogeneity by these factors (e.g. trial-level factors can be explored using everything mentioned in tutorial/in the MLM and nod to references for appropriate analyses for patient-level factors – making it clear that trial-level analysis is more straightforward, but patient-level analysis is trickier and might need additional statistical support to get right)
   2. A quiz with 2-3 questions, asking readers to sort factors into: trial-level, patient-level or either. Examples of some of the factors we can mention are:
      - Age, Sex, BMI (patient-level – but be careful of trial-level means)
      - Trial-design, Blinding status of outcome assessors, Trial setting (trial-level)
      - Treatment dose, Disease severity (either – depends on the trial design)
2. **Subgroup analysis for a categorical factor**
   1. A medium-length video (approx. 10-12 mins)
      - Describing subgroup analysis for a binary factor with a worked example, with points on interpretation
      - Describing a test for subgroup differences and how to use this alongside the subgroup effects
      - Extending to a categorical factor with three categories with a worked example
      - Briefly mentioning how to extend to more than three categories
      - Describing what to do with continuous factors
   2. Simple code to implement this in Stata and R
   3. A quiz with 4-5 interpretation questions, covering the following made-up situations and asking readers what these subgroup analyses mean:
      - Example 1: Subgroup1 crosses the null, Subgroup 2 does not cross the null. No test for subgroup differences carried out.
      - Example 2: Subgroup 1 does not cross null. Subgroup 2 crosses the null. A non-significant test for subgroup differences (e.g. p=0.3).
      - Example 3: Quantitative but not qualitative interaction.
      - Example 4: Quantitative & qualitative interaction.
      - Example 5: Three-categories, how to interpret. Choose a reference category and express contrasts with respect to that reference category (noting that the choice of reference in arbitrary)
3. **Meta-regression**
   1. A medium-length video (approx. 10-12 mins)
      - Why might you want to carry out meta-regression?
      - Meta-regression is just an extension of trial-level subgroup analysis
      - Meta-regression with a single categorical factor walkthrough (same factor as in 2), interpreting what the results mean – including what the intercept means (estimated effect when covariates are all zero – does this make sense? If not, may wish to centre the covariates first)
      - Meta-regression with a single continuous factor walkthrough (geographical latitude example), interpreting what the results mean
      - Meta-regression with multiple factors
      - Caveats to think about with meta-regression (lack of data per covariate, only explaining trial-level heterogeneity, not getting at patient-level variation)
   2. Simple code to implement this in Stata and R
   3. A quiz with 4-5 interpretation questions covering the following made-up situations and asking readers what these meta-regression results mean:
      - Non-significant categorical factor
      - Significant categorical factor
      - Non-significant continuous factor
      - Significant continuous factor
      - Multiple factors – interpret both/all
4. **Key tips when exploring heterogeneity using trial-level characteristics**
   Just a quiz, with some prompting questions for each of the four tips. These tips are paraphrased from tutorial 2 for ease.
   1. Tip 1: A large number of studies are needed before heterogeneity can be explored reliably using trial-level characteristics.
      - Q1: 5 studies in the meta-analysis, 4 in one group, 1 in the other. Can heterogeneity by this trial-level factor be explored reliably?
      - Q2: 18 studies in the meta-analysis, 7 in one group, 11 in the other. Can heterogeneity by this trial-level factor be explored reliably?
   2. Tip 2: Do not compare effect estimates in different subgroups by considering the meta-analysis results from each subgroup separately.
      - Q3: Example where one subgroup crosses the null and the other does not, but no test for subgroup differences has been done. Ask what the interpretation is. Answer: need to work out the test for subgroup differences.
      - Q4: Quantitative and qualitative interaction, similar to earlier quiz question, but double-checking interpretation here. Mention ICEMAN here in the answer when referring to what else they might do to assess whether this is a genuine subgroup difference.
   3. Tip 3: There should be a clear rationale for the inclusion of subgroup characteristics
      - Q5: Factor was pre-specified with a clear biological rationale for dose to impact the treatment effect, with a significant subgroup difference. Do you trust this result?
      - Q6: Analysis was post-hoc (not pre-planned), 10 factors have already been explored, and there is no clinical rationale. Do you trust this result?
   4. Tip 4: Considering a characteristic that varies within a trial as a trial-level characteristic could introduce bias.
      - Q7: Mean age is considered as a factor for meta-regression. Are there any risks with doing this?
      - Q8: Mean BMI is used to split trials into subgroups. Are there any risks with doing this?
